# KCa3.1 channel inhibition sensitizes malignant gliomas to temozolomide treatment

**DOI:** 10.18632/oncotarget.8761

**Published:** 2016-04-16

**Authors:** Giuseppina D'Alessandro, Alfonso Grimaldi, Giuseppina Chece, Alessandra Porzia, Vincenzo Esposito, Antonio Santoro, Maurizio Salvati, Fabrizio Mainiero, Davide Ragozzino, Silvia Di Angelantonio, Heike Wulff, Myriam Catalano, Cristina Limatola

**Affiliations:** ^1^ Department of Physiology and Pharmacology, Sapienza University of Rome, Rome, Italy; ^2^ IRCCS Neuromed, Pozzilli, Italy; ^3^ Department of Molecular Medicine, Sapienza University of Rome, Rome, Italy; ^4^ Department of Neurology and Psychiatry, Sapienza University of Rome, Rome, Italy; ^5^ Department of Science and Medical Surgical Biotechnology, Sapienza University of Rome, Rome, Italy; ^6^ Department of Experimental Medicine, Sapienza University of Rome, Rome, Italy; ^7^ Center for Life Nanoscience, Istituto Italiano di Tecnologia@Sapienza, Rome, Italy; ^8^ Department of Pharmacology, University of California Davis, Davis, USA; ^9^ Pasteur Institute Rome-Department of Physiology and Pharmacology, Sapienza University of Rome, Rome, Italy

**Keywords:** Ca^2+^ activated K^+^ channels, malignant glioma, apoptosis, cell cycle, migration

## Abstract

Malignant gliomas are among the most frequent and aggressive cerebral tumors, characterized by high proliferative and invasive indexes. Standard therapy for patients, after surgery and radiotherapy, consists of temozolomide (TMZ), a methylating agent that blocks tumor cell proliferation. Currently, there are no therapies aimed at reducing tumor cell invasion. Ion channels are candidate molecular targets involved in glioma cell migration and infiltration into the brain parenchyma. In this paper we demonstrate that: i) blockade of the calcium-activated potassium channel KCa3.1 with TRAM-34 has co-adjuvant effects with TMZ, reducing GL261 glioma cell migration, invasion and colony forming activity, increasing apoptosis, and forcing cells to pass the G2/M cell cycle phase, likely through cdc2 de-phosphorylation; ii) KCa3.1 silencing potentiates the inhibitory effect of TMZ on glioma cell viability; iii) the combination of TMZ/TRAM-34 attenuates the toxic effects of glioma conditioned medium on neuronal cultures, through a microglia dependent mechanism since the effect is abolished by clodronate-induced microglia killing; iv) TMZ/TRAM-34 co-treatment increases the number of apoptotic tumor cells, and the mean survival time in a syngeneic mouse glioma model (C57BL6 mice implanted with GL261 cells); v) TMZ/TRAM-34 co-treatment reduces cell viability of GBM cells and cancer stem cells (CSC) freshly isolated from patients.

Taken together, these data suggest a new therapeutic approach for malignant glioma, targeting both glioma cell proliferating and migration, and demonstrate that TMZ/TRAM-34 co-treatment affects both glioma cells and infiltrating microglia, resulting in an overall reduction of tumor cell progression.

## INTRODUCTION

Malignant gliomas (III grade astrocytoma and IV grade glioblastoma (GBM) according to the WHO classification) are the most diffuse and aggressive neoplasia of the central nervous system, characterized by high proliferation, angiogenesis, suppression of immune responses and invasiveness. Despite current treatments consisting of surgery followed by adjuvant radiotherapy and chemotherapy with the methylating agent temozolomide (TMZ), GBM patients display a median overall survival of only 14.6 months [1]. Tumors invariably recur, mainly due to residual cancer stem cells (CSC) [2] and to malignant cells escaping surgery and infiltrating healthy parenchyma. Even though significant advances in cancer molecular targeting have been made, several promising preclinical results revealed poor translatability (www.clinicaltrial.gov). TRAM-34 is a high affinity blocker of KCa3.1, exhibiting good selectivity for these channels [3]. In the healthy adult brain, KCa3.1 channels are primarily expressed in microglia [4] and vascular endothelial cells [5] and are mostly undetectable in other cell populations [6]. In contrast, in GBM, KCa3.1 channels are also functionally expressed by tumor cells [7–9]. The REepository of Molecular BRAin Neoplasia DaTa (REMBRANDT) database identified the gene KCNN4, which encodes for KCa3.1, as being over-expressed in 32% of glioma patients and correlating with significant shortened survival [10]. We and others recently demonstrated the involvement of KCa3.1 channels in GBM cell spreading in healthy parenchyma [10, 11]. Considering that malignant gliomas consist of proliferating and migrating cells [12–14], with the last often exhibiting reduced sensitivity to anti-proliferative or pro-apoptotic drugs [15, 16], we wondered whether KCa3.1 inhibition or silencing could potentiate the effects of standard chemotherapy with TMZ. TMZ is a cytotoxic imidazotetrazine that leads to the formation of O^6^- methylguanine, which mismatches with thymine in subsequent DNA replication cycles, with effects on several cellular functions, such as apoptosis [17], autophagy [18], mitotic catastrophe and senescence-like events [17]. In most cells, TMZ produces cell cycle arrest in the G2/M phase, through the activation of the DNA damage checkpoint pathways ATM/ATR-Chk1/2/cdc25C [19, 17]. G2/M check point activation requires cyclin B binding and phosphorylation of cdc2 at Thr14 and Tyr15 [20, 21]. Forcing cells to escape this check point might induce mitotic catastrophe and cell senescence [17, 22–24].

TRAM-34 is safe and well tolerated in various animal models [25]; it crosses the blood brain barrier, reaching biologically active concentrations in the brain parenchyma [11, 26]. In this paper we co-treated GBM cells, CSC acutely isolated from patients, and glioma cell lines with TMZ and TRAM-34, describing co-adjuvant effects on different parameters like cell invasion, proliferation, and apoptosis, as well as viability. TMZ treatment of KCa3.1 silenced glioma cells reproduced several effects of TRAM-34/TMZ co-treatment. TRAM-34 and TMZ co-treatment of glioma bearing mice also increased the frequency of apoptotic cells and significantly increased the mean survival time.

## RESULTS

### Combination of TRAM-34 and TMZ reduces migration and invasion of GL261 cells

**Figure 1:**
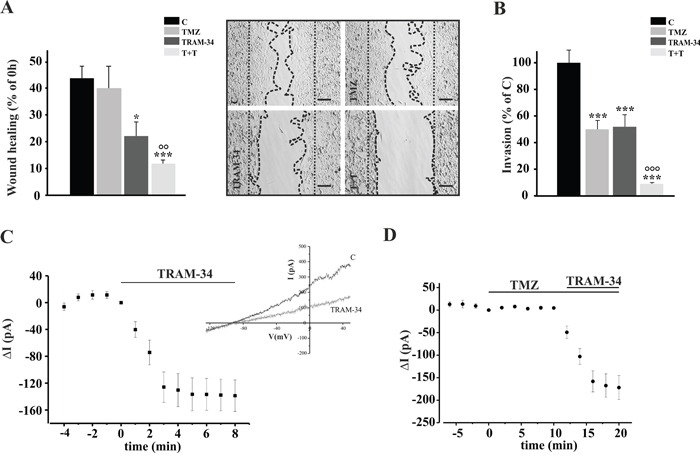
TRAM-34/TMZ treatment reduces basal cell migration and invasion of GL261 cells **A.** GL261 cells were treated with vehicle (control, C), TMZ, TRAM-34 or both (T+T) for 48 h and assayed for migration. The wound healing was analyzed using ImageJ software and data are expressed as [1- (empty area at 48 h/ empty area at 0 h)] x100, where the empty area at 0 h is indicated by the dotted parallel lines and the empty area at 48 h is comprised between the dotted curved lines. *p<0.05; ***p<0.001 *vs* C; °° p<0.01 *vs* TMZ n=4, One-Way ANOVA, Student-Newman-Keuls post-test. Representative picture of wound healing assay is shown on the right (4X magnification, bar = 100 μm). **B.** GL261 cells were treated as in **A** and plated on Matrigel film for 48 h; ***p<0.001 *vs* C; °°° p<0.001 vs TMZ n=4, One-Way ANOVA, Student-Newman-Keuls post-test. **C.** Time course of the effect of TRAM-34 (2.5 μM) on current evoked in GL261 cells (n= 12) by repeated voltage ramps (from −130 mV to + 50 mV, holding potential −70 mV). Acute application of TRAM-34 revealed the functional expression of KCa3.1 channels. Typical current trace in response to repeated ramps is shown in the inset. **D.** Time course of the effect of TMZ (30 μM) application alone and in co-application with TRAM-34 (2.5 μM) on current evoked in GL261 cells (n= 7) by repeated voltage ramps.

To determine whether the basal migration and invasion across extracellular matrix of GL261 cells were modulated by KCa3.1 channel inhibition with TRAM-34, by TMZ, or both, wound healing and transwell matrigel invasion assays were performed. TMZ and TRAM-34 concentrations were chosen as specified in the Methods. Figure 1A shows cell movement in a wound healing assay upon drug treatment: after 48 h, untreated cells covered 45% of wounded area, similarly to TMZ treated cells. TRAM-34 reduced the wound healing ability (to 22% ± 5) and the combination TMZ/TRAM-34 further reduced basal cell migration (to 12% ± 1). Figure 1B shows that TRAM-34 and TMZ treatment (48 h) significantly reduced GL261 cell invasion through a Matrigel layer, and that the co-treatment resulted in a stronger reduction.

To investigate whether the effect of TMZ on cell movement could be due to a direct effect on KCa3.1 channel activity, patch clamp recording of GL261 cells was performed in the presence of TMZ. We observed that KCa3.1 channels were functional in GL261 and their activation was not affected by TMZ (Figure 1C–1D). Conversely, KCa3.1 activity did not modify the membrane resting potential of these cells, which was −38.7 ± 4.3 mV in untreated and −38.5 ± 6.3 mV in TRAM-34 treated cells (n= 26).

### TRAM-34 and TMZ co-treatment reduces colony formation and proliferation of GL261 cells

**Figure 2:**
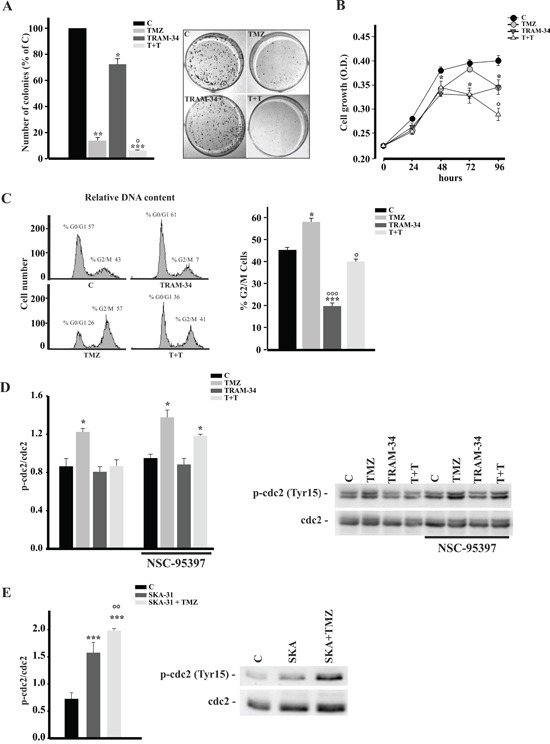
TRAM-34/TMZ treatment reduces clonogenicity and growth of GL261 cells and alters cell cycle **A.** GL261 cells were treated with vehicle (control, C), TMZ, TRAM-34 or both (T+T) for 24 h. Colonies grown were counted at 14 days. Data are expressed as % of C, *p<0.05, **p<0.01 and ***p<0.001 *vs* C; ° p<0.05 *vs* TMZ n=4 by One-Way ANOVA, Student-Newman-Keuls post-test. Right, representative picture of a colony forming assay. **B.** Growth curve of GL261 cells treated as in A for the indicated time points. The results are expressed as function of the optical density (OD) at 590 nm.*p<0.05*vs* C; ° p<0.05 *vs* TMZ n=3, One-Way ANOVA, Student-Newman-Keuls post-test. **C.** GL261 cells were treated as in **A** for 72 h and cell cycle analysis was performed by flow cytometry. Representative experiment on the left shows % cells in different phases of the cell cycle. The mean percentage of cells in G2/M phase with the different treatments is shown on the right. *p<0.05 and ***p<0.001 *vs* C; ° p<0.05 and °°° p<0.001 *vs* TMZ n=3 by One-Way ANOVA, Student-Newman-Keuls post-test. **D.** GL261 cells were treated as in **A** in absence or presence of NSC-95397(60 nM) for 48 h, lysed and analyzed by Western Blot for cdc2 phosphorylation. Data are shown as p-cdc2/cdc2 ratio; *p <0.05*vs* C n = 4, One-Way ANOVA, Student-Newman-Keuls post-test. Right, representative blot. **E.** GL261 cells were treated with vehicle (control, C), SKA-31 (250 nM) or SKA-31 + TMZ (30 μM) for 48 h, lysed and analyzed by Western Blot for cdc2 phosphorylation. Data are shown as p-cdc2/cdc2 ratio; ***p <0,001 *vs* C n = 4,°° p <0,01 *vs* SKA-31. One-Way ANOVA, Student-Newman-Keuls post-test. Right, representative blot.

We wondered whether combined treatment with TRAM-34 and TMZ of glioma cells could reduce tumor cell proliferation more efficiently than TMZ alone. Towards this aim, the effect of TRAM-34 and TMZ was tested on clonogenicity and proliferation of GL261 using a colony forming assay and performing a growth curve staining cells with crystal violet. As shown in Figure 2A, TRAM-34 alone had a smaller but significant effect, in comparison with TMZ, on the number of colonies, as expected from the known TMZ sensitivity of GL261 cells [27]. Interestingly, combined TMZ/TRAM-34 treatment further reduced colony growth. With the same method, we also tested the proliferation of GL261 cells treated with TMZ and TRAM-34 or both. As shown in Figure 2B, all treatments reduced cell growth, but again the combined TMZ/TRAM-34 treatment further decreased cell proliferation, with induction of cell death at 96 h. Data on cell proliferation were also confirmed using a MTT assay (Supplementary Figure S1). These results indicate an increased cell sensitization to TMZ upon KCa3.1 inhibition and prompted us to investigate the effect on GL261 cell cycle upon single or combined drug treatments. Cell cycle distribution were investigated by FACS and western blot analyses: Figure 2C shows that GL261 cells treated with TRAM-34 have an increased frequency in G0/G1, further confirmed by a decreased expression of cyclin D1 (Supplementary Figure S2). TMZ treatment induced cell arrest in G2/M phase, as already observed in other cells [22, 23], while the combined TMZ/TRAM-34 treatment overrode the effect of TMZ on cell cycle arrest. Since cdc2 activation by phosphorylation on Tyr15 blocks cells from entering in mitosis [20], we analyzed the effect of TMZ, TRAM-34 or both on cdc2 phosphorylation. Figure 2D shows that, upon TMZ treatment, cdc2 phosphorylation increased, while TRAM-34 co-treatment blocked protein activation. Of note, TRAM34 alone was ineffective on cdc2 phosphorylation. To further validate the effect of KCa3.1 block on cdc2 modulation, GL261 cells were treated with TRAM-34 in the presence of a specific phosphatase cdc25 inhibitor, NSC95397. Under these conditions, TRAM-34 did not block TMZ effect (Figure 2D), demonstrating that cdc25C is involved. In contrast, a specific KCa3.1 channel activator (SKA-31) increased cdc2 phosphorylation and SKA-31/TMZ co-treatment further increased it (Figure 2E), indicating that channel function might sustain cell cycle arrest also upon TMZ-induced DNA damage. Taken together, these data demonstrate that KCa3.1 blockade with TRAM-34 counteracts the effect of TMZ on cell cycle arrest and further reduces tumor cell proliferation.

### TRAM-34/TMZ co-treatment increases apoptosis in glioma cells

**Figure 3:**
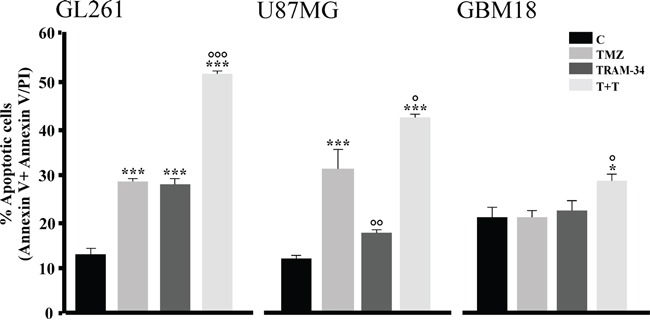
TMZ/TRAM-34 treatment increases apoptosis in murine and human malignant glioma cells GL261, U87MG and GBM18 cells were treated with vehicle (control, C), TMZ, TRAM-34 or both (T+T) for 96 h. Cell apoptosis was detected by flow cytometry; data represent the mean value of Annexin V positive plus Annexin V/PI positive cells expressed as percentage of total cells. *p<0.05 and ***p<0.001 *vs* C; ° p<0.05, °° p<0.01 and °°° p<0.001 *vs* TMZ n=3 by One-Way ANOVA, Student-Newman-Keuls post-test.

To test if the effect of TRAM-34 and TMZ treatment on cell cycle could result in tumor cell apoptosis, glioma cells were treated with TRAM-34, TMZ or both and stained with AnnexinV/PI. The data in Figure 3 show that, at 96 h, TMZ/TRAM-34 significantly increased the number of GL261 apoptotic cells in comparison with TRAM-34 and TMZ alone. TMZ/TRAM-34 treatment also increased apoptosis in other glioma cells like the human U87MG and the patient-derived GBM (GBM18), suggesting common effects on apoptotic pathways. As already shown for GL261 cells, we found functional expression of KCa3.1 channels by patch clamp recordings also in the human GBM cell tested (Supplementary Figure S3).

### TMZ treatment decreased viability of KCa3.1 silenced glioma cells

**Figure 4:**
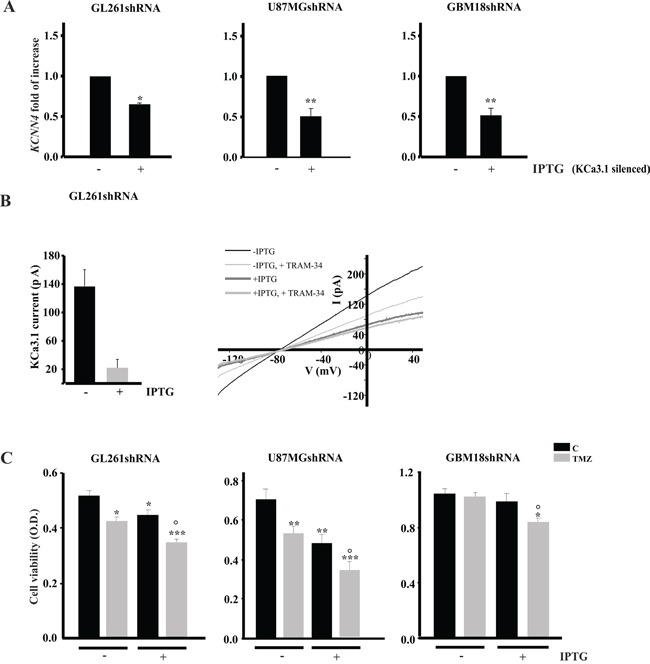
KCa3.1 silencing and TMZ treatment reduce glioma cell viability **A.** GL261, U87MG and GBM18 cells were infected with IPTG-inducible shRNA construct to silence KCa3.1 channel (GL261shRNA, U87MGshRNA and GBM18shRNA). Cells were induced (+IPTG) or not (−IPTG) and assayed for the expression of channel mRNA (*KCNN4*) by RT-PCR. Data are expressed as fold increase *vs* -IPTG. *p<0.05 and **p<0.01 *vs* –IPTG. n=4 by One-Way ANOVA, Student-Newman-Keuls post-test. **B.** Bar chart showing average KCa3.1 current amplitude, obtained as TRAM-34 (2.5 μM) sensitive current, in GL261shRNA cells without (n=7) and with IPTG induction (n=8), by repeated voltage ramps (from −130 mV to + 50 mV, holding potential −70 mV). Right, typical current trace in response to repeated ramps in control (black trace) and in induced GL261shRNA (grey trace). **C.** GL261shRNA, U87MGshRNA and GBM18shRNA cells were treated with vehicle (C) or TMZ for 96 h and tested for cell viability by MTT assay. Cell viability is expressed as a function of optical density (OD). *p<0.05, **p<0.01 and ***p<0.001 *vs* C; ° p<0.05 *vs* TMZ n=4 by One-Way ANOVA, Student-Newman-Keuls post-test.

To verify that the effect of TRAM-34 was due to its specific inhibition of KCa3.1 activity, GL261, U87MG and primary glioblastoma cells (GBM18) were silenced for KCa3.1 expression with IPTG-inducible shRNA constructs, treated with TMZ and analyzed for viability. The efficacy of silencing was confirmed by RT-PCR, and shown in Figure 4A, with reductions of 35.3±1.7% (GL261), 47.8±8.9% (U87MG) and 50.0±7.5% (GBM18) of KCNN4 mRNA upon IPTG induction. GL261shRNA cells were also tested for KCa3.1 current: patch clamp recordings performed on vehicle- and IPTG-induced GL261 cells demonstrated that KCa3.1 currents were strongly reduced in these cells (Figure 4B), thus supporting the efficacy of channel silencing. TMZ treatment (96h) of silenced cells significantly reduced cell viability, similarly to what was observed for TRAM-34/TMZ co-treatment (Figure 4C). Note that in GBM18shRNA, only TMZ/TRAM-34 co-treatment reduced cell viability, comparably to what was reported for Annexin V/PI staining (Figure 3).

### TRAM-34 reduces neuronal death induced by GL261 cells acting through microglia

**Figure 5:**
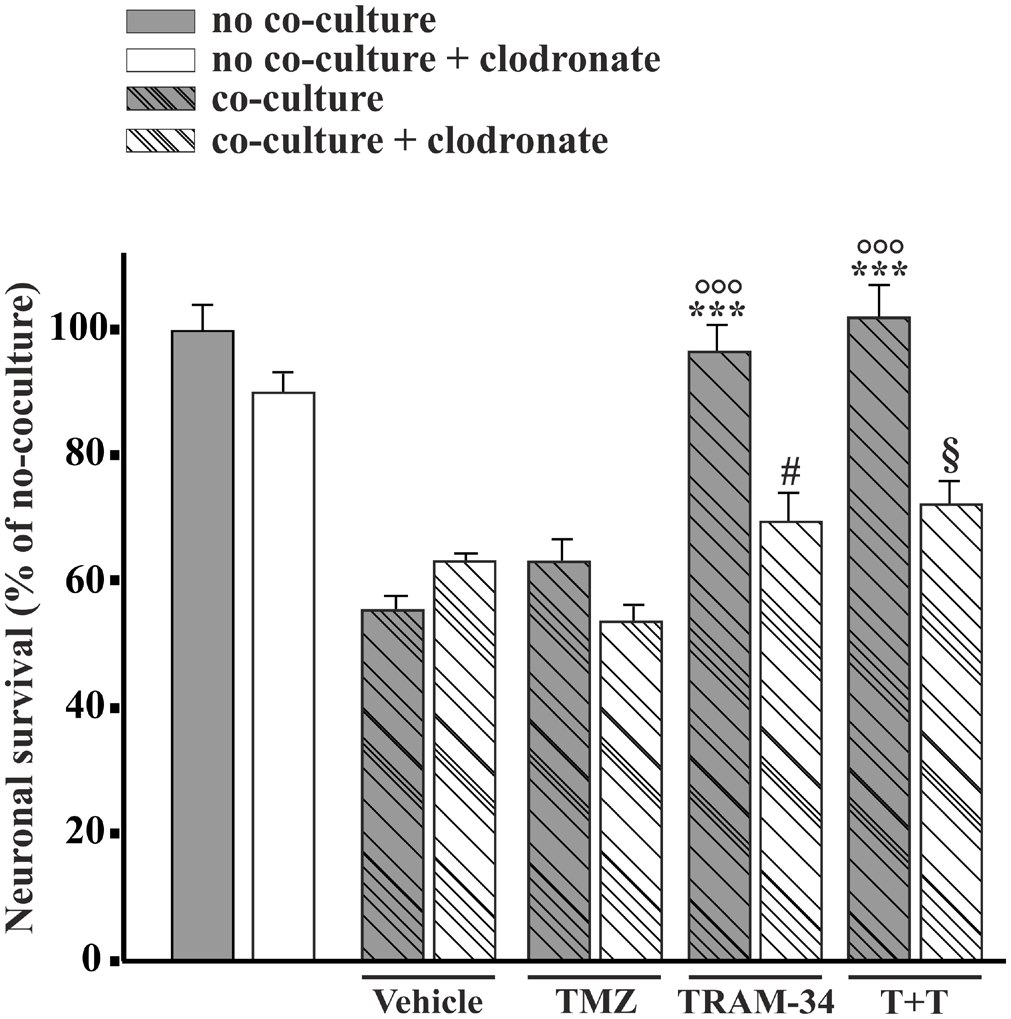
Microglia cells mediate TRAM-34-induced neuroprotection against GBM toxicity Hippocampal cultures, treated with empty (grey bars) or clodronate-filled liposomes (white bars) were co-cultured or not (C) with GL261 cells and treated with vehicle, TMZ, TRAM-34 or both (T+T). Results are expressed as percentage of cell survival, taking C as 100%. ***p<0.001; # p<0.05; § p<0.05; °°° p<0.001; n=3, One-Way ANOVA, Student-Newman-Keuls post-test.

To investigate whether KCa3.1 inhibition also affects the brain tumor microenvironment, we co-cultured primary hippocampal cells (60–70% neurons, 30-35% astrocytes, 4–8% microglia) with GL261 in the presence or absence of TRAM-34, TMZ or both. As expected, under these conditions, hippocampal neurons died from excitotoxicity [28] induced by glutamate released by GL261 (293 ± 2μM in the medium after 24 h in culture). TRAM-34 and TMZ/TRAM-34 treatments significantly reduced neuronal death, while TMZ alone was ineffective (Figure 5). Neuroprotection was measured with two different independent tests with similar results (data reported refer to the cell lysis protocol, see methods). Since KCa3.1 channels are also expressed by microglia, we evaluated the microglia involvement in the neuroprotective effect of TRAM-34 by their specific depletion with clodronate-containing liposomes. Results shown in Figure 5 indicate that the absence of microglia abolished the neuroprotective effects of TRAM-34, highlighting a key intermediate role for this cell population in reducing neuronal toxicity.

### TRAM-34/TMZ co-treatment increases survival in GL261 bearing-mice, reduces tumor volume and increases apoptosis

**Figure 6:**
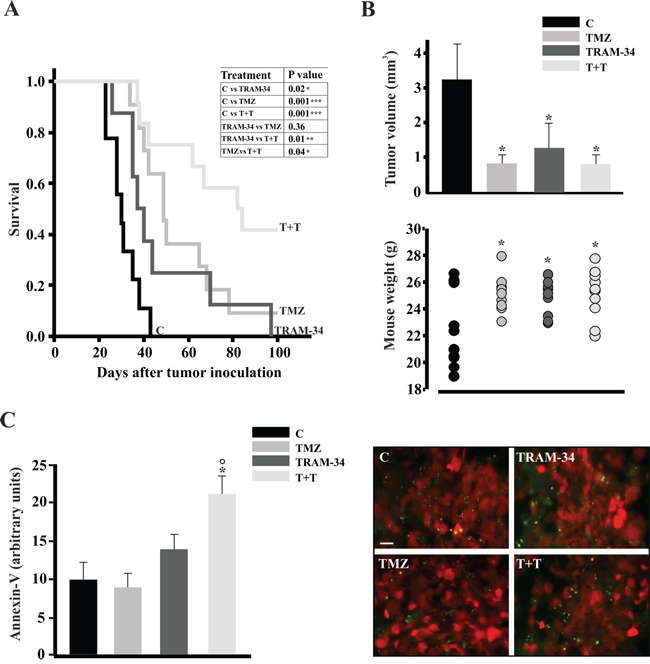
TRAM-34 and TMZ/TRAM-34 treatment increases survival of GL261-bearing mice **A.** Kaplan-Meier survival curves of glioma bearing mice treated with vehicle (control, C), TRAM-34, TMZ or both (T+T) (n=8-11, log-rank test results are shown in the inset). **B.** Glioma bearing mice, 21 days after glioma cell inoculation, were analyzed for tumor volumes (upper panel; * p<0.05 *vs* C; n= 7, One-Way ANOVA, Student-Newman Keuls post-test) and mouse weight (lower panel; *p<0.05 *vs* C; n=10-11, One-Way ANOVA, Student-Newman-Keuls post-test). **C.** Glioma bearing mice, 21 days after glioma cell inoculation, were analyzed for apoptosis. Brain slices (tumor core) obtained from mice injected with GL261-RFP cells (red) treated with vehicle (control, C), TRAM-34, TMZ or both (T+T) analyzed for immunofluorescence of Annexin V (green, bar =10 μm); results are expressed as arbitrary units obtained by the ratio between Annexin-V positive area and tumor area analyzed; * p<0.05 *vs* C; ° p<0.05 *vs* TMZ n= 3, One-Way ANOVA, Student-Newman Keuls post-test.

The results obtained *in vitro* prompted us to investigate the possible effects of TRAM-34 and its combination with TMZ in a syngeneic glioma mouse model. Figure 6A illustrates the survival of mice-injected with GL261 cells upon TRAM-34, TMZ or TMZ/TRAM-34 treatment. All conditions significantly increased mice survival but, again, the combination of TRAM-34 and TMZ was more effective in term of mean survival time (76± 7 days) in comparison with TMZ (55 ± 6days) and TRAM-34 (48 ± 8days) alone (survival time of control animals was 31± 2 days). Beneficial effects of treatments were already observed after 21 days as decrease in tumor volume (Figure 6B, upper panel) and increase of body weight (Figure 6B, lower panel). These mice also had a higher proportion of apoptotic cells in the tumor mass: Figure 6C shows that GL261-RFP-bearing mice treated with TMZ/TRAM-34 had increased Annexin-V positive cells (green) in the tumor core (red cells) in comparison with vehicle-treated or TRAM-34 and TMZ treated mice after 21 days. The effects on tumor volume and apoptosis could underlie the increase in survival observed in TMZ/TRAM-34 treated mice.

### TRAM-34/TMZ treatment decreases cell viability of GBM cells and CSC

**Figure 7:**
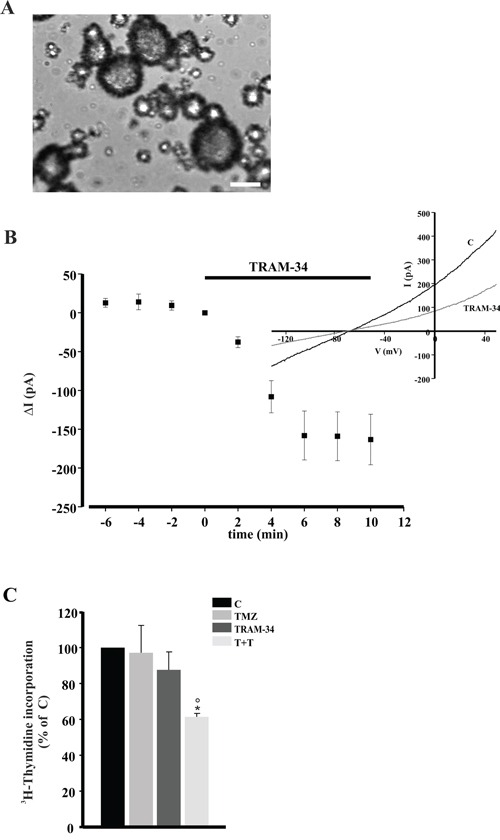
TRAM-34/TMZ treatment decreases CSC proliferation **A.** Representative morphology of GBM enriched CSC cultured as neurospheres. Magnification 10X. Scale bar 500 μm **B.** Time course of the effect of TRAM-34 (2.5 μM) on current evoked in GBM enriched CSC cells (n= 10) by repeated voltage ramps (from −130 mV to + 50 mV, holding potential −70 mV). Acute application of TRAM-34 revealed the functional expression of KCa3.1 channels. Typical current trace in response to repeated ramps is shown in the inset. **C.** CSC were treated for 4 days with TMZ, TRAM-34 or both (T+T) and analyzed for proliferation measured as [^3^H]-thymidine incorporation. Data are expressed as % of vehicle-treated cells (control, C); * p<0.05 *vs* C; ° p<0.05 *vs* TMZ n= 3, One-Way ANOVA, Student-Newman Keuls post-test.

To address the potential validity of a future clinical use of a KCa3.1 blocker such as TRAM-34 combined with TMZ, we tested the effect of the combination on GBM cells acutely obtained from fourteen patients and on CSCs obtained from one patient. Table 1 shows cell viability, measured by MTT assay, upon 5 day treatment with TMZ or in combination with TRAM-34 in comparison with untreated cells (C). At least in seven samples (GBM14, GBM19, GBM29, GBM33, GBM55, GBM109, GBM111), TMZ/TRAM-34 treatment significantly reduced cell viability in comparison with TMZ alone, with more scattered results in the others. DNA synthesis, measured as [^3^H]-thymidine incorporation, was tested in enriched-CSC cultures, maintained as neurospheres (Figure 7A). CSC were preliminary assayed for CD133 expression, and KCa3.1 channel expression, showing 20% of CD133+ cells in the preparation (Supplementary Figure S4). These cells had TRAM-34 sensitive KCa3.1 currents (Figure 7B). Following four day treatment with TMZ, TRAM-34 or both, we observed that only TRAM-34/TMZ co-treatment significantly reduced DNA synthesis (Figure 7C).

**Table 1:** Effect of TRAM-34 and TMZ treatment on the viability of GBM cells from patients

	CELL VIABILITY (% of C)	
	TMZ	T+T
*GBM9*	105 ± 3	108 ± 2
*GBM14*	102 ± 5	85 ± 2 ^# *^
*GBM19*	102 ± 2	89 ± 2 ^## *^
*GBM20*	92 ± 3^*^	90 ± 1^*^
*GBM29*	102 ± 3	88 ± 2^## *^
*GBM33*	78 ± 3 ^**^	71 ± 1^# **^
*GBM44*	97 ± 5	112 ± 2^# *^
*GBM55*	94 ± 1	78 ± 1 ^### **^
*GBM73*	85 ± 1^**^	111 ± 4 ^##^
*GBM107*	103 ± 2	119 ± 8 ^#^
*GBM109*	107 ± 2	94 ± 1 ^###^
*GBM110*	94 ± 5	88 ± 1^*^
*GBM111*	93 ± 3	82 ± 3 ^## *^
*GBM112*	103 ± 4	100 ± 4

GBM cells acutely dissociated from patients were treated for 5 days with TMZ and TMZ/TRAM-34 (T+T) and analyzed for viability by MTT assay. Results are expressed as viable cells in treated vs untreated samples (%). * p<0.05, ** p<0.01 *vs* C; # p<0.05, ##p<0.01, ###p<0.01 *vs* TMZ, One-Way ANOVA, Student-Newman-Keuls post-test analysis.

Abbreviation: KCa3.1, intermediate-conductance calcium-dependent potassium channel 1; TRAM-34, 1-[(2-chlorophenyl)(diphenyl)methyl]-1*H*-pyrazole; GBM, glioblastoma multiforme; TMZ, temozolomide; DMEM, Dulbecco's modified minimum essential medium; FBS, fetal bovine serum; MTT, 3-(4, 5-Dimethylthiazol-2-yl)- 2, 5-diphenyltetrazolium bromide; GFAP, glial fibrillary acidic protein; BSA, bovine serum albumin; HEPES, hydroxyethyl piperazineethanesulfonic acid; DMSO, dimethyl sulfoxide; PBS, potassium phosphate buffer; ANOVA, analysis of variance; SKA-31, naphtho [1, 2-*d*] thiazol-2-ylamine.

Taken together these data confirm the ability of TRAM-34 to sensitize cells obtained from GBM patients to TMZ cytotoxicity. The reason for the failure to increase TMZ cytotoxicity in about 50% of samples remains to be established but could be due to individual variability, also for KCa3.1 over-expression [10].

## DISCUSSION

New therapeutic strategies aiming to fight both migrating and proliferating glioma cells are necessary to effectively counteract GBM progression. In the present study we describe that glioma-bearing mice, when co-treated with TRAM-34 and TMZ, significantly increase the mean survival time in comparison with single-drug treatments. The beneficial effect of TMZ/TRAM-34 co-treatment in glioma bearing mice can be explained by the following effects observed *in vitro*: i) reduced glioma cell migration and invasion; ii) reduced colony forming ability, glioma proliferation rate and apoptotic index; iii) alterations of cell cycle progression; iv) protection from glioma-induced neuronal cell death. Similar results were obtained with cells acutely obtained from patients and in GBM- enriched CSC, since TMZ/TRAM-34 also reduced their viability.

GBM recurrence in TMZ treated patients is often due to diffuse glioma invasiveness of cerebral parenchyma. Recent data suggest key roles for ion channels in mediating tumor cell migration [29], with direct involvement of calcium-activated potassium channels. We and others have recently demonstrated that KCa3.1 inhibition, or silencing, reduces tumor cell infiltration in the brain parenchyma in experimental mouse models of the disease [10, 11], thus supporting previous *in vitro* findings [30]. We now demonstrated that KCa3.1 inhibition or silencing significantly enhances the anti-proliferative effects of the alkylating agent TMZ, increasing the number of apoptotic cells and reducing cell viability, suggesting KCa3.1 as a key therapeutic target for glioma. We demonstrate that the combined treatment with TMZ/TRAM-34 reduces tumor cell infiltration and migration more potently than single treatment. We also show that TMZ/TRAM-34 decreased glioma cell proliferation, reducing cell survival and clonal ability. These effects are likely due to the increased apoptosis, induced through decreased phosphorylation level of proteins regulating cell cycle, like cdc2. Activation of cdc2 is regulated by a balance between kinases (cdk) and phosphatase (cdc25C) activity [31]. It is known that TMZ and other DNA-damaging agents inactivate cdc25C, arresting cells in G2, where cells check and repair possible DNA copy errors [17, 32]. Ion channels have often been associated with cell cycle progression through their involvement in the shape changes required for cell division [33–35]. We show that TMZ/TRAM-34 co-treatment forces cells to exit the G2 checkpoint and to move forward the G0/G1 phases, a process that induces mitotic catastrophe, cell senescence, and apoptotic death [22]. We observed a significant increase of apoptotic, Annexin V/PI positive cells upon TMZ/TRAM-34 treatment, both *in vivo*, in glioma injected mice and *in vitro*, in glioma cell lines. Other molecules involved in cell cycle regulation, such as 7-hydroxystaurosporine, indolocarbazole inhibitor (SB-218078) and resveratrol, oppose to TMZ-induced cell arrest in G2 phase [17, 23, 24]. KCa3.1 in particular is required for cell cycle transition to the S phase and entry into G2/M, in different cancer cells [36, 37], and is involved in irradiation-induced cell accumulation in G2/M [38]. We demonstrated that cdc25C is a possible intermediate of the effect of TMZ/TRAM-34 on glioma cell death [17].

Data obtained in GBM cell lines were in part confirmed in human tumor cells obtained from patients. In these cells we observed that TMZ/TRAM-34 treatment significantly reduced cell viability in comparison with TMZ-treated cells in 7 out of 14 specimens. In addition, we observed that TMZ/TRAM-34 treatment significantly reduced proliferation of CSC obtained from one GBM patient, further validating this therapeutic approach.

It is known that microglia and macrophages invading GBM play key roles in tumor progression, representing the largest population of tumor-infiltrating cells [39]. We have previously shown that KCa3.1 inhibition reduced astrogliosis and microglia activation in glioma bearing mice [11] and reduces microglia activation and infarct size, increasing neuronal survival in a mouse model of ischemic stroke [26]. Glioma cells induce neuronal death through glutamate release in the extracellular space [28]. Here we show that TRAM-34 reduces GL261 cells-induced neuronal death in a co-culture system. Specific depletion of microglial cells from the hippocampal cultures with clodronate, abolished TRAM-34 induced neuronal protection, demonstrating a specific effect of KCa3.1 expressed on microglia in reducing glioma-induced neuronal cell death. We have recently demonstrated that the blockade of KCa3.1 activity on microglia conditioned by glioma modifies their phenotype to a pro-inflammatory, anti-tumor state [40]. However, we cannot exclude that in the tumor microenvironment this microglia phenotype also induces a direct protective effect on neurons [41].

Together, these data pave the road to the understanding of the molecular mechanisms involved in the increase of survival time in TMZ/TRAM-34 treated mice. In particular TRAM-34 increases TMZ mediated cytotoxicity, reduces tumor cell invasion, and reduces neuronal cell death induced by glioma (summarized in Supplementary Figure S5). Therefore, KCa3.1 inhibition in combination with TMZ may offer a new therapeutic approach for GBM patients. This approach is attractive since TMZ is the first line chemotherapeutic agent (and often the only one) used for this disease, and KCa3.1 overexpression in patients correlates with a poor prognosis [10, 42, 43]. In addition the KCa3.1 blocker Senicapoc (ICA-17043), which is structurally related to TRAM-34, has already been used in clinical trials and has been found to be safe for patients [44, 45].

## MATERIALS AND METHODS

### Materials

Cell culture medium (Dulbecco's modified minimum essential medium, DMEM), fetal bovine serum (FBS), penicillin G, streptomycin, glutamine, sodium pyruvate and Hoechst 33342 were from GIBCO Invitrogen (Carlsbad, CA); rabbit anti p-cdc2(Tyr15) was from Cell Signaling (Danvers, MA), mouse anti cdc2p34 and cyclin D1 were from Santa Cruz (Dallas, TX); Matrigel and Transwell inserts were from BD (Franklin Lakes, NJ); Encapsome and Clodrosome were from Encapsula NanoSciences (Nashville, TN); 3-(4, 5-Dimethylthiazol-2-yl)- 2, 5-diphenyltetrazolium bromide (MTT) salt, DMSO, peanut oil, SKA-31, pLKO.1 lentiviral shRNA clones targeting human and murine KCa3.1 mRNA and all other chemicals were from Sigma-Aldrich (St. Louis, MO) or Pierce (Rockford, IL). TRAM-34 was synthesized in our lab as previously described [3]. NSC95397 was from Enzo Life Sciences, Inc. (New York, NY).

### Experiments with animals

Experiments described in the present work were approved by the Italian Ministry of Health in accordance with the guidelines on the ethical use of animals from the European Community Council Directive of 22 September 2010 (2010/63/EU). All efforts were made to minimize the number of animals used and their suffering. We used C57BL/6 mice from Charles River Laboratories.

### Cell cultures

GL261 cells were kindly provided by Dr. Serena Pellegatta, Neurological Institute “Carlo Besta”, Italy. U87MG, GL261 and GL261-RFPcells (obtained as previously described, [46]) were cultured in DMEM supplemented with 10-20% heat-inactivated FBS, 100 IU/ml penicillin G, 100 μg/ml streptomycin, 2.5 μg/ml amphotericin B, 2 mM glutamine, and 1 mM sodium pyruvate. Cells were grown at 37°C in a 5% CO_2_ humidified atmosphere. Cells were subcultivated when confluent.

Human freshly dissected and primary GBM cells (GBM18) were obtained after patient surgery at Policlinico Umberto I (Rome) and Neuromed (Pozzilli, IS), from GBM patients who gave a written informed consent to the research proposals. The study was approved by the Institutional Ethics Committee of Sapienza University. Histopathological typing and tumor grading were done according to the WHO criteria resulting as grade IV. GBM cells from patients were obtained as described in our previous work [30]. After 10-15 days, adherent cells were sub-cultured once and then used for experiments. Freshly dissociated cell cultures were named as described in Table 1. Primary GBM18 were obtained after further culturing adherent tumor cells and were used between passages 20 and 30.

### Cancer stem cells

Glioblastoma enriched CSC culture was established from freshly dissociated surgical specimen as previously described [47]. After three weeks, cells in suspension grew as neurospheres. For the experiments, the spheres were dissociated to single cells suspension, for no more than three times.

### Glioma cell treatments

Before treatments, cells were shifted to culture medium containing 1% FBS, vehicle (DMSO), TMZ (30 μM), TRAM-34 (5 μM) or both. The TMZ concentration was chosen considering the levels reported for patients treated with standard therapy [48–50]. The TRAM-34 concentration was chosen based on previous experiments [11, 30] and based on test experiments (MTT proliferation assay) demonstrating that 5 μM TRAM-34 was necessary to increase TMZ effect (Supplementary Figure S6). Final DMSO concentration was always 0.08% (v/v). Incubation time was specified for each experiment.

### Wound-healing assay

Cells (3.5 × 10^4^) were seeded and cultured into the inner wells of cell culture inserts (ibidi, Germany) and placed in a Petri dish. Once attached to the substratum, the inserts were removed from the surface leaving a 500 μm cell-free wound. To evaluate basal migration, the wounded areas were photographed at 0 and 48h with a CoolSNAP camera (Photometrics) coupled to an ECLIPSE Ti-S phase contrast microscope (Nikon, Japan) and processed using MetaMorph 7.6.5.0 image analysis software (Molecular Device, CA). Wound healing was measured as follows: [1- (empty area at 48h/ empty area at 0h)] x100.

### Transwell invasion assay

Sub-confluent GL261 cells were trypsinized, and plated in invasion medium (DMEM supplemented with 100 IU/ml penicillin G and 100 μg/ml streptomycin, 0.1% BSA and 25 mM HEPES, pH 7.4), at a density of 7 × 10^3^ cells/cm^2^ on matrigel-coated transwells (Corning, 8 μm pore size). Cells were treated and incubated for 48 h at 37°C, then fixed in ice-cold 10% trichloroacetic acid for 10 min. Cells adhering to the upper side of the filter were scraped off, whereas cells invaded through the insert were stained with a solution containing 50% isopropanol, 1% formic acid and 0.5% (w/vol) brilliant blue R 250 (Sigma-Aldrich) and counted in at least 20 fields with a 20x objective.

### Colony forming assay

GL261 cells were trypsinized, counted and plated (1 × 10^3^) in 6 cm dishes containing 20% FBS medium. After 6h the growth medium was changed and supplemented with TRAM-34 or TMZ for 24h. Every three days the medium was changed and only TRAM-34 was refreshed; 14 days after plating cells were fixed in 4% p-formaldehyde in PBS and stained with cresyl violet for 20 min. Only colonies containing more than 50 cells were scored as positive.

### Glutamate release

Glutamate concentration was determined in cellular supernatant by reversed-phase High Performance Liquid Chromatography (HPLC) following Dabsyl Chloride derivatization [51].

### Protein preparation and western blot analysis

For protein analysis, 5 × 10^5^ GL261 cells were seeded on 12 well plates and treated with vehicle, TMZ, TRAM-34 or both for 48h; cells were washed with PBS and lysed in hot 2x Laemmli buffer, boiled 5 min and sonicated. The same amount of proteins was separated on 12% SDS-polyacrylamide gel and analyzed by Western immunoblot using the following primary antibodies: cyclin D1 1:200, p-cdc2 (Tyr15) 1:1000, cdc2p34 1:200. HRP-tagged goat anti-rabbit and anti mouse-IgG were used as secondary antibodies (1:2000; Dako), and detection was performed by the chemiluminescent assay Immun-Star WesternC Kit (Bio-Rad, CA). Densitometric analysis has been carried out with Quantity One software (Biorad, CA).

### Primary hippocampal neuronal cultures

Primary hippocampal neuronal cultures were prepared from 0-2-day old (p0–p2) C57BL/6 mice. Briefly, after careful dissection from diencephalic structures, the meninges were removed and hippocampal tissues chopped and digested for 20 min at 37°C in 0.025% trypsin and Hank's balanced salt solution (HBSS). Cells were washed twice with HBSS to remove the excess of trypsin, mechanically dissociated in minimal essential medium (MEM) with Earl's Salts and GLUTAMAX supplemented with 10% dialyzed and heat inactivated FBS. Cells were plated at a density of 2 × 10^5^ in the same medium on poly-L-lysine- (100 μg/ml) coated plastic 24-well dishes. After 2h, the medium was replaced with serum- free Neurobasal/B27. Cells were kept at 37°C in 5% CO2 for 11 days. With this method we obtained 60-70% neurons, 30-35% astrocytes, 4-8% microglia, as determined with β-tubulin III, GFAP, and IBA-I staining [52].

### Neurons/GL261-RFP co-cultures

Primary hippocampal neuronal cultures, at the 9^th^ day in culture, were treated with empty or clodronate encapsulating liposomes (Cl_2_MBP) [52] to deplete microglial cells. After 24h, cells were co-cultured with or without GL261-RFP (8 × 10^5^), for 18h on 6.5mm transwells (Corning, 0.4 μm pore size).

### Neuronal viability assay

To evaluate neuron viability, hippocampal neuronal cultures were treated with detergent-containing buffer (0.05% ethyl hexadecyl dimethylammonium bromide, 0.028% acetic acid, 0.05% Triton X-100, 0.3 mM NaCl, 0.2 mM MgCl2, in PBS pH 7.4) and viable nuclei counted in a hemacytometer as described [53, 54]. Neuronal viability was also evaluated by MTT assay.

### Growth curves, cell cycle analysis and apoptosis

GL261 cells (3.5 × 10^4^) were seeded into 12-well plates in triplicates and after 6h treated with TRAM-34, TMZ or both. For growth curves, after 1-4 days, cells were rinsed twice with PBS, fixed for 15 min in PFA 4% and stained with 0.1% crystal violet. Acetic acid (10%) was used to dissolve the stain. Absorbance was measured at 590 nm and results were expressed as mean optical density (OD) ± SE. For cell cycle analysis, GL261 cells were harvested with trypsin-EDTA, washed with cold PBS, fixed in 70% ethanol for 1 h at 4°C and stained with 50 μg/ml propidium iodide (PI) supplemented with 250 μg/ml RNase A for 30 min at RT in the dark. DNA content was measured using a FACSCalibur (BD Biosciences, NJ). For the detection of apoptosis, cells were harvested, washed with Binding Buffer (10 mM HEPES, 140 mM NaCl, and 2.5 mM CaCl_2_), and resuspended in FITC-conjugated Annexin V (Bender MedSystems, Austria). After 15 min of incubation at RT, PI was added, and the percentage of AnnexinV-FITC and Annexin V-FITC/PI^+^ cells was determined.

### KCa3.1 silencing by lentiviral transduction of shRNA constructs

GL261, U87MG and primary GBM18 cells were infected by lentivirus directing IPTG-inducible expression of KCa3.1 shRNA. Cells (1.6 × 10^4^) were plated in 96-well plates and infected for 24 h according to the manufacturer's instructions. Transduced cells were selected with 2.5 μg/ml puromycin for 3–12 days. IPTG (5 mM) was added to culture medium to induce shRNA expression. Knockdown efficiency of KCa3.1 channels was evaluated by real time PCR (RT-PCR) and electrophysiological recordings. Cells were then named GL261shRNA, U87MGshRNA and GBM18shRNA.

### Real time PCR

GL261shRNA, U87MGshRNA and GBM18shRNA cells (7 × 10^5^) were induced with 5 mM IPTG or vehicle for 5 days. Total RNA was extracted by Trizol reagent (Invitrogen), quantified using the Ultraspec 2000 UV/Visible (Pharmacia Biotech) and the reverse transcription reaction was performed in a thermocycler (MJ Mini Personal Thermal Cycler; Biorad) using IScriptTM Reverse Transcription Supermix (Biorad) according to the manufacturer's protocol. RT-PCR was carried out in a I-Cycler IQ Multicolor RT-PCR Detection System (Biorad) using Sso Fast Eva Green Supermix (Biorad) according to the manufacturer's instructions. The PCR protocol consisted of 40 cycles of denaturation at 95°C for 30 s and annealing/extension at 58°C for 30 s. For quantification analysis the comparative Threshold Cycle (Ct) method was used. The Ct values from each gene were normalized to the Ct value of GAPDH in the same RNA samples. Relative quantification was performed using the 2^−ΔΔCt^ method and expressed as fold increase in arbitrary values. Primer sequences for genes are: mouse *gapdh* forward 5′-3′ TCGTCCCGTAGACAAAATGG, reverse 3′-5′ TTGAGGTCAATGAAGGGGTC; human *GAPDH* 5′-3′ forward CCCCTTCATTGACCTCAACTAC; 5′-3′ reverse GATGACAAGCTTCCCGTTCTC; mouse *kcnn4* forward 5′-3′ GGCTGAAACACCGGAAGCTC reverse 3′-5′ CAGCTCTGTCAGGGCATCCA; human *KCNN4* forward 5′-3′ GGCTGAAACACCGGAAGCTC reverse 3′-5′ CAGCTCTGTCAGGGCATCCA.

### MTT cell viability assay

Acutely isolated GBM cells, GBM and glioma cell lines, and silenced cells were seeded into multi-well plates and treated with vehicle (C), TMZ, TRAM-34 or TMZ/TRAM-34 for 4 or 5 days. MTT (500 μg/ml) was added into each well for 1.5 h. DMSO was then added to stop the reaction and the formazan produced was measured at 570 nm. Viability of cells was expressed relative to absorbance.

### [^3^H]-Thymidine incorporation assay

GBM enriched CSCs (500 cells/well) were seeded in 96-well round-bottomed microtest culture plates and treated with vehicle, TRAM-34, TMZ or both. After 4 days of incubation, cells were pulsed with 1μCi of [^3^H]-thymidine per well for the last 18 h, harvested and counted. Tests were performed in triplicate and results were expressed as the percentage of vehicle treated cells.

### Intracranial inoculation

Eight week-old male mice were anesthetized with chloral hydrate (400 mg/kg, i.p.) and placed in a stereotaxic head frame. Animals were injected with 1 × 10^5^ GL26-RFP cells: a median incision of ~1 cm was made, a burr hole was drilled in the skull, and cells were injected in the right striatum (−2 mm lateral, +1 mm antero-posterior from Bregma). Cell suspension in PBS (5 μl) was injected with a Hamilton syringe at a rate of 1 μl/min at 3 mm depth. After 7 days mice were treated with TRAM-34 (120 mg/kg/daily i.p. in peanut oil), TMZ (50 mg/kg i.p. every two days for four times with two weeks stop) or both. After 21 days or at moribund state, mice were sacrificed. The 21 day period was necessary to complete a TMZ cycle. Animals used in Kaplan-Meier survival studies received up to four TMZ cycles.

### Tumor volume measurement and immunostaining

Brains were isolated and fixed in 4% buffered p-formaldehyde 21 days after GL261-RFP injection. Coronal brain sections (20 μm) were prepared by standard procedures and stained with hematoxylin and eosin. A section every 80 μm was collected, and the tumor volume was evaluated using Image Tool 3.00. To detect apoptosis, parallel serial brain sections were washed in PBS, blocked (3 % goat serum in 0.03 % Triton X-100) for 1 h at RT, and incubated overnight at 4°C with FITC-conjugated Annexin V. Apoptosis was measured under a fluorescence microscope as the area occupied by Annexin V-positive cells versus the analyzed area; this pixel-based method was chosen to evaluate the scattered Annexin V signal. At least 6 coronal sections per brain were analyzed.

### Patch-clamp experiments

Cells (GL261, U87MG, GBM18, CSC, GL261shRNA) were patched in the whole-cell configuration. Micropipettes (4–5 MΩ) were filled with an intracellular solution containing the following (in mM): K-Asp 145, MgCl_2_ 2, HEPES 10, EGTA 10, CaCl_2_ (3 μM free Ca^2+^, calculated using the MaxChelator software: http://www.stanford.edu/~cpatton/maxc.html, pH 7.3 adjusted with KOH, osmolarity 290 mOsm; Sigma Aldrich). During experiments, glioma cells were continuously superfused with an extracellular solution based on Na-Aspartate containing (in mM): Na-Asp 160, KCl 4.5, CaCl_2_ 2, MgCl_2_ 1, HEPES 10 (pH 7.3), using a valve-controlled gravity driven perfusion system (VC-6 Warner Instruments) connected to a perfusion pencil multi-barrel manifold tip (100 μM diameter, Automate Scientific), for standard and antagonist-containing solutions. All recordings were performed at 24–25°C. Voltage-clamp recordings were carried out using an Axopatch 200B amplifier (Molecular Devices). Currents were filtered at 2 kHz, digitized (10 kHz) and collected using Clampex 10 (Molecular Devices); the analysis was performed off-line using Clampfit 10 (Molecular Devices). The current/voltage (I/V) relationship of each cell was determined applying voltage ramps from −130 to +50 mV for 200 ms; the holding potential was −70 mV between ramps. KCa3.1 current expression was evaluated using TRAM-34 (2.5 μM, 8 min); in particular, it was the result of the algebraic subtraction of the current obtained after TRAM-34 application, from the control current. As glioma cells display a prominent Kv current, all experiments were performed in the presence of the Kv1.3 blocker PAP-1 (1 μM).

### Statistics

All data shown as mean values with standard errors were analyzed with One Way ANOVA by Student-Newman-Keuls post test. Mouse survival was compared between groups with the Kaplan-Meier survival analysis with log-rank test.

## SUPPLEMENTARY MATERIALS FIGURES


