# Supplementary material for: KCa3.1 channel inhibition sensitizes malignant gliomas to temozolomide treatment
Source: Oncotarget. 2016 Apr 16;7(21):30781–96. doi: 10.18632/oncotarget.8761 (PMC5058717; doi:10.18632/oncotarget.8761)
Supplement: Supplementary file 1 [file oncotarget-07-30781-s001.pdf]

KCa3.1 channel inhibition sensitizes malignant gliomas to temozolomide treatment

SUPPLEMENTARY METHODS

FACS analyses

To evaluate the percentage of CD133 positive cells, neurospheres were mechanically dissociated, incubated with (293C3)-APC antibody (Myltenyi Biotec) or with equivalent amount of IgG2b-APC isotype control for 15 minutes in the dark and analyzed by flow cytometry.

SUPPLEMENTARY FIGURES

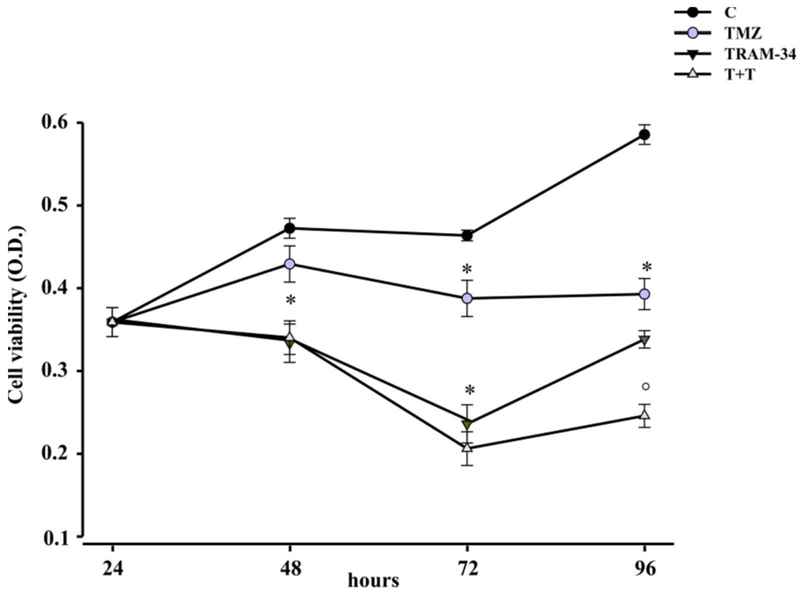

Supplementary Figure S1: TRAM-34/TMZ treatment reduces cell viability of GL261 cells. GL261 cells were treated with vehicle (C), TMZ (30 microM), TRAM-34 (5 microM) or both (T+T) for the indicated time points and tested for viability by MTT assay. Viability is expressed as function of optical density (OD) at 590 nm, *p<0.05 *vs* C; ° p<0.05 *vs* TMZ n=4 by One-Way ANOVA, Student-Newman-Keuls post-test.

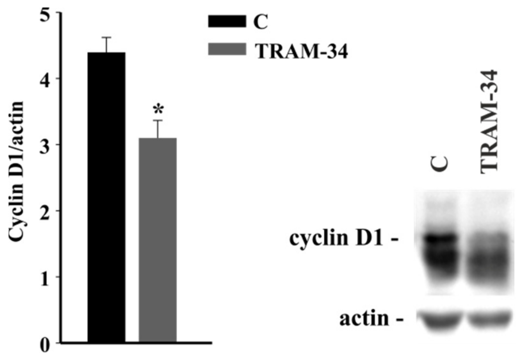

Supplementary Figure S2: TRAM-34 treatment reduces cyclin D1 expression. GL261 cells were treated with vehicle (C) or TRAM-34 (5 microM) for 72 h and analyzed for cyclin D1 expression by Western Blot. Actin was used as loading control. Data are expressed as cyclin D1/actin ratio.*p <0.05 *vs* C n = 3, Students' *t*-test. Right, representative blot.

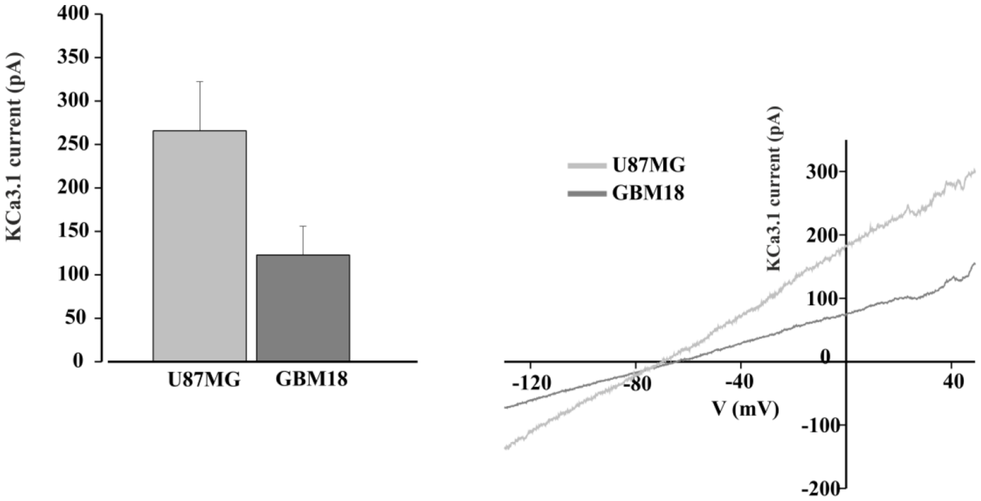

Supplementary Figure S3: Functional expression of KCa3.1 channels in U87MG and GBM18 cells. Bar chart showing average KCa3.1 current amplitude (pA), obtained as TRAM-34 (2.5 µM) sensitive current, in U87MG (n=9) and GBM18 (n=6) cells by repeated voltage ramps (from -130 mV to + 50 mV, holding potential -70 mV). Right, typical current trace in response to repeated ramps in U87MG and GBM18 cells.

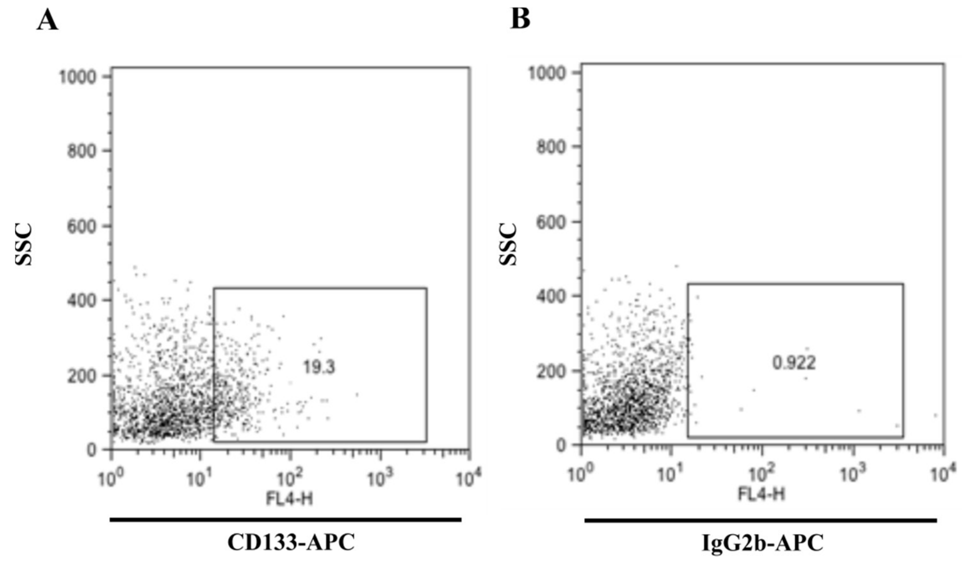

Supplementary Figure S4: Characterization of GBM enriched CSC by CD133 staining. A. Dot plot of CD133 positive cells. **B.** IgG2b-APC was the isotype control.

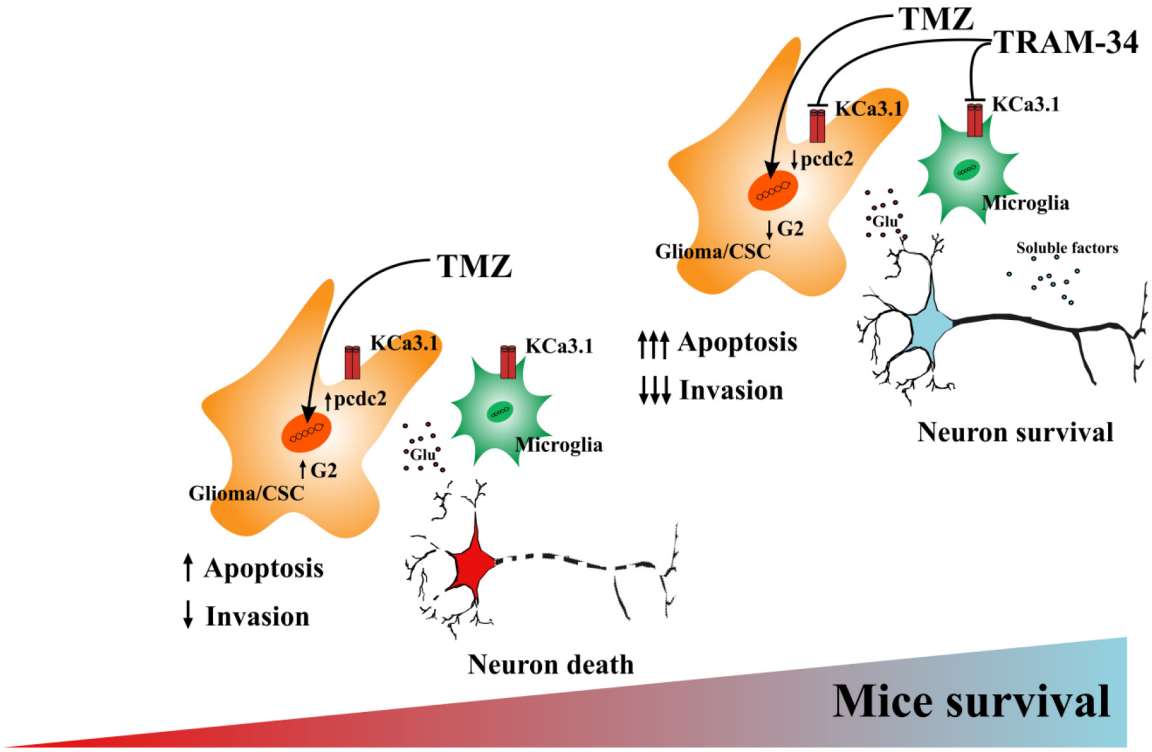

Supplementary Figure S5: Summary scheme of the effects induced by TRAM-34/TMZ co-treatment. Treatment with TMZ of mice injected with glioma reduced tumor growth, increasing cell apoptosis and reducing glioma infiltration. TMZ/TRAM34 co-treatment strongly increased TMZ mediated apoptosis, decreasing cdc2 phosphorylation and the number of cells in G2 phase, and further reduced cell invasion. TRAM-34 also promoted neuroprotection against GBM neurotoxicity with mechanisms involving microglial cells. The overall effect of TRAM-34/TMZ co-treatment is an increase of TMZ-induced mice survival.

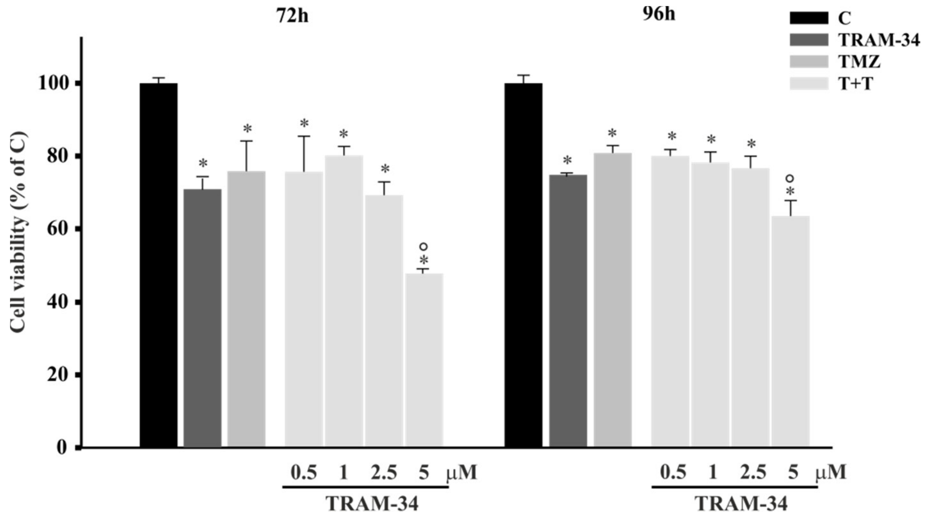

Supplementary Figure S6: TRAM-34/TMZ treatment reduces cell viability of GL261 cells. GL261 cells were treated with vehicle (C), TMZ (30 μM), TRAM-34 (5 μM) or both (T+T) (TRAM-34 concentrations are indicated) for 72 and 96 h and tested for viability by MTT assay. Data are expressed as % of C, *p<0.05*vs* C; ° p<0.05 *vs* TMZ n=4 by One-Way ANOVA, Student-Newman-Keuls post-test.
